# Efficacy of extracorporeal shock wave therapy and nutraceutical supplementation in the treatment of lateral epicondylitis: a randomized controlled trial

**DOI:** 10.3389/fresc.2025.1593909

**Published:** 2025-06-11

**Authors:** Dalila Scaturro, Domenico Migliorino, Sofia Tomasello, Michele Vecchio, Antimo Moretti, Giovanni Iolascon, Giulia Letizia Mauro

**Affiliations:** ^1^ Department of Medicine of Precision in the Medical, Surgical and Critical Care Areas, University of Palermo, Palermo, Italy; ^2^ Section of Pharmacology, Department of Biomedical and Biotechnological Sciences, University of Catania, Palermo, Italy; ^3^ Villa Delle Ginestre, Palermo Provincial Health Authority, Palermo, Italy; ^4^ Department of Medical and Surgical Specialties and Dentistry, University of Campania “Luigi Vanvitelli”, Naples, Italy

**Keywords:** nutraceuticals Rehabilitation, musculoskeletal disease, tendinopathy, tennis elbow, ultrasonography

## Abstract

**Introduction:**

Numerous scientific papers have compared different treatment options in the management of lateral epicondylitis. Our study evaluated the efficacy of focal extracorporeal shock wave treatment (ESWT) combined with an integrative nutraceutical treatment of hyaluronic acid, collagen, vitamin C, and manganese, compared with single treatment in patients with lateral epicondylitis in terms of improvement of pain, functional capacity, muscle strength, and reduction of inflammation on ultrasound images.

**Methods:**

A single-center, randomized controlled trial (RCT) was conducted in a population of patients with lateral epicondylitis. Patients were enrolled and randomly divided into 3 groups: Group A, consisting of 15 patients who were treated with twenty sessions of therapeutic exercise and five focal ESWT sessions (one session every six days); Group B, consisting of 15 patients who performed twenty sessions of therapeutic exercise and took daily a dietary supplement consisting of Hyaluronic Acid 200 mg, Collagen 5,000 mg, Vitamin C 250 mg and Manganese 10 mg for thirty days; and Group C, consisting of 15 patients, who received a combined treatment of therapeutic exercise, focal ESWT and nutraceutical supplementation The Numerical Rating Scale (NRS) and Patient-Rated Tennis Elbow Evaluation Scale (PRTEE) were administered during the first physiatric examination (T0), at 15 days after the start of treatment (T1), and at a 30-day follow-up (T2). In addition, muscle strength (Handgrip strength) was analyzed by means of a dynamometer, and common extensor tendon thickness (CET Thickness) by ultrasound examination.

**Results:**

Data analysis showed that NRS scale scores decreased significantly only in group B at T1 (*P* < 0.05), and in the three groups at T2 (*P* < 0.05). Grip strength increased significantly after 30 days only in group C (*P* < 0.05), while PRTEE scores and CET Thickness decreased significantly at T2 in the three groups (*P* < 0.05). The comparison among the various groups also showed that the Group C showed statistically significant improvements of function and grip strength at T2, compared with the other groups (*P* < 0.05).

**Discussion:**

Our study demonstrated that the combination of focal ESWT, therapeutic exercise, and nutraceutical supplementation, represent a viable therapeutic option for the management of lateral epicondylitis; likewise, the proposed treatments resulted in a synergistic effect for pain relief and functional recovery in the short term, providing a decrease in the inflammatory state and an increase in muscle strength.

## 1 Introduction

Lateral epicondylitis (LE), also known as “tennis elbow,” is a pathologic condition of the musculotendinous system characterized by pain and tenderness at the lateral epicondyle of the humerus (1, 2). It is a tendinopathy of the forearm extensor muscles, often resulting from overuse, repetitive arm movements, forced elbow extension, or direct trauma to the humeral epicondyle (3, 4). LE is a common condition affecting the common extensor tendon (CET), which originates from the fibers of the extensor carpi radialis brevis (ECRB), extensor digitorum, and extensor carpi ulnaris muscles, inserting at the lateral epicondyle of the elbow. The condition affects 1%–3% of the general population, primarily between the ages of 30 and 70, with no significant gender predisposition (5). Histologically, LE exhibits degenerative tendon changes, including fibroblast proliferation, vascular hyperplasia, and disorganized collagen (6). While the natural course of the condition is generally favorable, with resolution often occurring within two years, recurrence after asymptomatic periods is common (7). This chronicity has significant functional and economic implications, as it can limit daily activities and occupational performance. The primary cause of LE is microtrauma at the origin of the extensor and supinator muscles of the forearm, typically resulting from sports or occupational activities involving repetitive flexion-extension and pronation-supination movements of the elbow. Additionally, heavy lifting and frequent wrist extensions against resistance contribute to the onset of the condition, leading to impaired functioning (8–10).

The ECRB is the most frequently affected muscle, although; the pronator teres and other wrist extensor muscles may also be involved (11). While repetitive use is widely accepted as a cause of microfissures and microlesions, the role of inflammation remains debated. Many studies suggest that LE is primarily a degenerative tendon disease rather than a purely inflammatory condition, recommending the use of terms “tendinosis” or “tendinopathy” instead of “epicondylitis” (12).

Several theories have been proposed to explain the pathophysiology of LE, but no definitive evidence has established the impact of sarcomere length and microanatomical characteristics of the ECRB muscle in the development of the condition. Some researchers hypothesize that a traction response plays a key role in LE due to the microanatomy of the ECRB. When elongated, the muscle undergoes sarcomere stretching, forming a functional traction angle that negatively impacts microcirculation, causing ischemic processes in muscle fibers and tendons. This results in increased synthesis of immature type III collagen, disruption of collagen continuity and loss of load. Neovascularization is then initiated, accelerating tendon degeneration and preventing proper healing, potentially leading to tendon rupture (13, 14).

Histological analysis of tissue specimens from patients with LE reveals macroscopic changes at the tendon origin, along with microscopic features such as vascular proliferation, hyaline degeneration, fibroblastic proliferation, and calcific debris- characteristic of degenerative tendinosis rather than an acute inflammatory process. The etiology of pain in LE has also been suggested to have a neurogenic component, as studies indicate the presence of nerve fibers reactive to neuropeptides such as substance P (SP) and calcitonin gene-related peptide (CGRP) (15).

Clinically, LE manifests as acute pain and functional limitation at the elbow, often radiating along the dorsal forearm to the wrist and hand (16). Pain intensity varies and negatively affects patients' quality of life, with common complaints including grip weakness and difficulty lifting small objects, leading to reduced autonomy in daily activities (17). Diagnosis is primarily clinical, based on a thorough history and physical examination (5). In some cases, imaging modalities such as musculoskeletal ultrasound (18, 19) and MRI (20–24) are used for differential diagnosis and to assess inflammatory or degenerative changes in the CET (25).

Ultrasound images are supportive of clinical evaluation in patients with lateral epicondylitis, as expressed by the international guidelines of the ISPRM/International Society of Physical and Rehabilitation Medicine- EURO-MUSCULUS/USPRM. High-frequency B-mode ultrasound probes, in fact relate the anatomical features of the lateral elbow and its different ultrasound patterns in patients diagnosed with lateral epicondylitis. In addition, integration with high-sensitivity color/power Doppler allows the evaluation of microcirculation and the presence of an acute inflammatory state, with characteristic pathological perfusion (26). The treatment of LE is heterogeneous, aiming to control pain, recover joint ROM and grip strength, normalize limb function, and prevent complications such as tendon injury (27). Conservative treatment is successful in 90% of cases (12, 28, 29), with surgery reserved for complex, and recurrent cases. Patients are advised to modify activities that exacerbate symptoms and correct improper movement patterns. The RICE protocol (rest, ice, compression and elevation) is commonly recommended in the initial stages to alleviate pain (30).

Cuff braces can significantly relieve pain by applying pressure to the forearm extensor muscles, reducing stress at the ECRB origin (31). Pharmacological treatment includes oral or topical NSAIDs for pain management and inflammation control, and nutraceutical supplements containing hyaluronic acid and collagen may support tendon healing (32, 33). Peritendinous corticosteroid infiltrations are also used, though repeated or improperly performed injections may lead to tendon rupture or muscle atrophy, necessitating ultrasound guidance (34, 35). Rehabilitation improves joint ROM and pain, with stretching and eccentric strengthening exercises for the wrist and elbow extensors being particularly beneficial (36–38). Elastic taping of the wrist extensor tendons and lateral epicondyle has also shown promising results (39–41). Various physical therapies, including ultrasound and focal shock waves, enhance circulation, elasticity, and metabolism through mechanical and thermal effects (42–45). Laser therapy is widely employed for its analgesic and anti-inflammatory properties (46, 47). Platelet-rich plasma (PRP) injections have gained attention for stimulating tendon repair by promoting neovascularization in the collagen matrix, accelerating healing (48, 49). Surgical intervention is reserved for patients with persistent pain and disability who do not respond to conservative treatment (4%–11% of cases). Surgical options include open, percutaneous, and arthroscopic techniques, which aim to debride the degenerated portion of the ECRB tendon, with or without wrist extensor tendon repair (50–54).

The aim of our study was to compare the efficacy of single and combined treatments- focal extracorporeal shock wave therapy (ESWT), and nutraceutical supplementation with hyaluronic acid, collagen, vitamin C, and manganese- in patients with LE in terms of pain reduction, improved functional capacity, increased muscle strength, and pathophysiological changes in tendon components as observed through ultrasound imaging.

## 2 Materials and methods

### 2.1 Study design

At the U.O.C. of Functional Recovery and Rehabilitation of the Policlinico “Paolo Giaccone” in Palermo, we conducted a single-center, non-blinded RCT on a population of patients with LE. We conducted a non-blinded study because we preferred to directly monitor and observe the effect of the treatments; likewise, participants expressed immediate feedback to us, facilitating the collection of qualitative data, net of an increased risk of observational bias and a placebo effect of the proposed treatments. The study was conducted between March 2024 and December 2024; for the data collection of this study, we included a consecutive series of patients, who were referred to the U.O.C. of Functional Recovery and Rehabilitation of the A.O.U.P. “Paolo Giaccone” of Palermo during the period between April 2024 and November 2024 to undergo physiatric evaluation. The study received the approval of the Local Ethics Committee “Palermo 1” (Approval No. 8/2024) and was conducted following the Declaration of Helsinki. The information and data were processed according to good clinical practice guidelines (GCP). The nutraceutical is duly registered in the register of dietary supplements of the Italian Ministry of Health. The compound was manufactured according to good manufacturing practice (GMP) standards to ensure constant control according to quality standards; product compliance was also monitored before administration. All subjects signed informed consent before inclusion, the study was conducted following the CONSORT guidelines for randomized controlled trials (RCTs); it was also registered on ClinicalTrials.gov (NCT06442618).

### 2.2 Participants

The selection criteria were as follows: age between 18 and 45 years, a diagnosis of LE with ultrasonographic evidence of inflammatory status of the CET of the wrist, a Numerical Rating Scale (NRS) at T0 ≥ 4, a pharmacological washout period beginning seven days before treatment, and written informed consent.

According to the EURO-MUSCULUS/USPRM guidelines, a tendon is defined as inflamed when structural changes (usually thickening) are followed by altered echogenicity (usually the tendon appears hypoechogenic) and inhomogeneous vascularization with local increase in inflammatory cytokines (often hypervascularization occurs). Interestingly, in patients with signs and symptoms of LE, a hypertrophic neurovascular network has been identified histologically within the aforementioned superficial soft tissues that surrounds and penetrates the superficial fibers of the CET. Among the various pathological conditions potentially involved in the clinical scenario of LE, focal tendinosis, partial tear, and intratendinous calcific deposition are the most commonly encountered in daily practice (26). Patients were excluded from the study if they were pregnant, had neoplasms, were pacemaker carriers, had coagulation disorders and/or were undergoing anticoagulant therapy, had skin lesions and/or local infections, had tendon lesions, had previously undergone wrist extensor tendon surgery, had cervical myelopathy, had epilepsy, had contraindications and/or allergies to the active ingredients of the nutraceutical supplementation, or had obesity (BMI > 30 kg/m^2^). Using our hospital database, we enrolled a consecutive series of patients with LE who were undergoing rehabilitation and met our inclusion criteria.

### 2.3 Intervention

We recruited a total of 45 patients with LE, who were randomly divided in a 1:1:1 ratio into three groups through a system of computer-generated random numbers; the division was therefore completely random without taking into account the data present at T0; group A received twenty sessions of therapeutic exercise and five focal ESWT sessions (one session every six days); group B performed two cycles of therapeutic exercise (20 sessions) and received daily nutraceutical supplemental for thirty days, containing hyaluronic acid (200 mg), collagen (5,000 mg), vitamin C (250 mg), and manganese (10 mg); group C received a combined treatment of therapeutic exercise, focal ESWT and nutraceutical supplementation with the same timing and modalities.

#### 2.4.1 A group (ESWT)

Participants in Group A attended our outpatient clinics in our department wearing comfortable clothing. They underwent daily rehabilitation sessions lasting 60 min, five days a week, for a total of twenty sessions. A physical therapist supervised exercises, including upper limb muscles, maximal eccentric contraction exercises for the wrist extensor muscles, progressive strengthening exercises (starting with isometric and progressing to dynamic exercises for the arm muscles), and grip strength exercises with individualized weight lifting. Additionally, patients received focal ESWT every six days for a total of five sessions, each lasting approximately 20 min. Treatment energy and frequency followed the International Society for Medical Shock Wave Treatment (ISMST) guidelines, with specific parameters (80–100 mJ with 2,250 pulses of 5–10 Hz). A physiatrist provided a 1:1 ratio of patient supervision, explained the treatment modality beforehand, and clinically evaluated the patient to identify the pain site before each session. Patient were positioned comfortably for treatment according to ISMST protocol (55).

#### 2.4.2 B group (nutraceutical supplementation)

Participants in Group B combined the same rehabilitation treatment as Group A, with daily nutraceutical supplementation for 30 days. The supplement contained hyaluronic acid (200 mg), collagen (5,000 mg), manganese (10 mg), and vitamin C (250 mg). Patient were instructed to take it at the same time each day and on an empty stomach to optimize absorption. They were also advised to shake the mixture before consumption and store it at a temperature below 25°C in a cool, dry place away from light and heat sources. The compound was gluten- and lactose-free, ensuring safe administration.

#### 2.4.3 C group (combined treatment)

Participants in group C underwent combined treatment, including therapeutic exercise, focal ESWT, and nutraceutical supplementation, following the same protocols as groups A and B.

### 2.5 Clinical evaluation

Demographic and clinical information was obtained from patients' medical records. The following assessments were conducted at three time points: baseline (T0), 5 days after treatment initiation (T1), and at a 30-day follow-up (T2): Pain intensity was measured using the Numerical Rating Scale (NRS), an 11-point scale ranging from 0 (no pain) to 10 (worst pain imaginable) (56–58); functional disability was assessed using the Patient-Rated Tennis Elbow Evaluation (PRTEE) Scale, which includes pain (5 items) and functional activity (10 items), each rated from 0 (no pain or difficulty) to 10 (worst pain or inability to perform tasks). The total score was the sum of both components (59–61); grip strength was measured using a Jamar Hydraulic Hand Dynamometer (Patterson Medical 081028935-IIN). Patients sat in a chair without armrests with shoulders in 0° abduction and neutral rotation, elbows at 90° flexion, and forearms in a neutral position. They performed three maximum-effort grips, each lasting 3 s, with a 60-second rest between trials. The average of the three attempts was recorded (62–64); CET thickness was assessed via ultrasound imaging using a linear probe (GE Healtcare Versana Essential – Linear Probe L3-12-RS). A blinded radiologist performed the assessments while patients sat with elbows flexed at 90°, wrists pronated, and arms resting on a table. The thickness and echogenicity of the CET and bony cortex of the lateral epicondyle were measured (65).

### 2.5 Statistical analysis

Data collection was performed using a spreadsheet (Microsoft Excel, version 16.58). The study was conducted following the CONSORT guidelines for randomized controlled trials (RCTs). We first calculated the sample size of the study, aiming to detect an average difference in the rating scales used between group A (ESWT), group B (nutraceutical supplementation) and group C (combined treatment). The sample size was 43 with a 99% confidence level and a margin of error of 5%, for all outcomes. We conducted a statistical power analysis using GPower software v. 3.1.9.4 and the powe size was 0,8. The score changes in the different rating scales were subjected to the distribution-based standard error of measurement (SEM) method to define clinical improvement. From this method, the minimum clinically important difference (MCID), defined as the smallest difference that patients and physicians perceive as useful, was presented for each instrument. Based on the SEM, a score change of 2.8 points in the NRS and 11 in the PRTEE corresponded to the MCID; the MCID value of grip strength was 5.3 points in males, and 4.2 points in females. On the other hand, as for the MCID of tendon thickness, it was corresponded to 0.48 points. It should be noted that the exact value of the MCID is not a fixed value and depends on the assessment method used to calculate the score change. The normality of the collected data was assessed using the Shapiro–Wilk test. Continuous variables were expressed as means and standard deviations, while categorical variables were reported as absolute numbers and percentages. The t-test was used to compare the means of quantitative variables. Finally, to compare the various treatments, we applied Tukey's HSD (honestly significant difference) procedure, which facilitates pairwise comparisons within the ANOVA data. The F statistic indicates whether there is an overall difference between the sample means, while Tukey's HSD test identifies which pairs of values, if any, differ significantly. Statistical analysis was conducted using R statistical software (R Core Team, 2021). Results with *p* ≤ 0.05 were considered statistically significant.

## 3 Results

**Figure 1:**
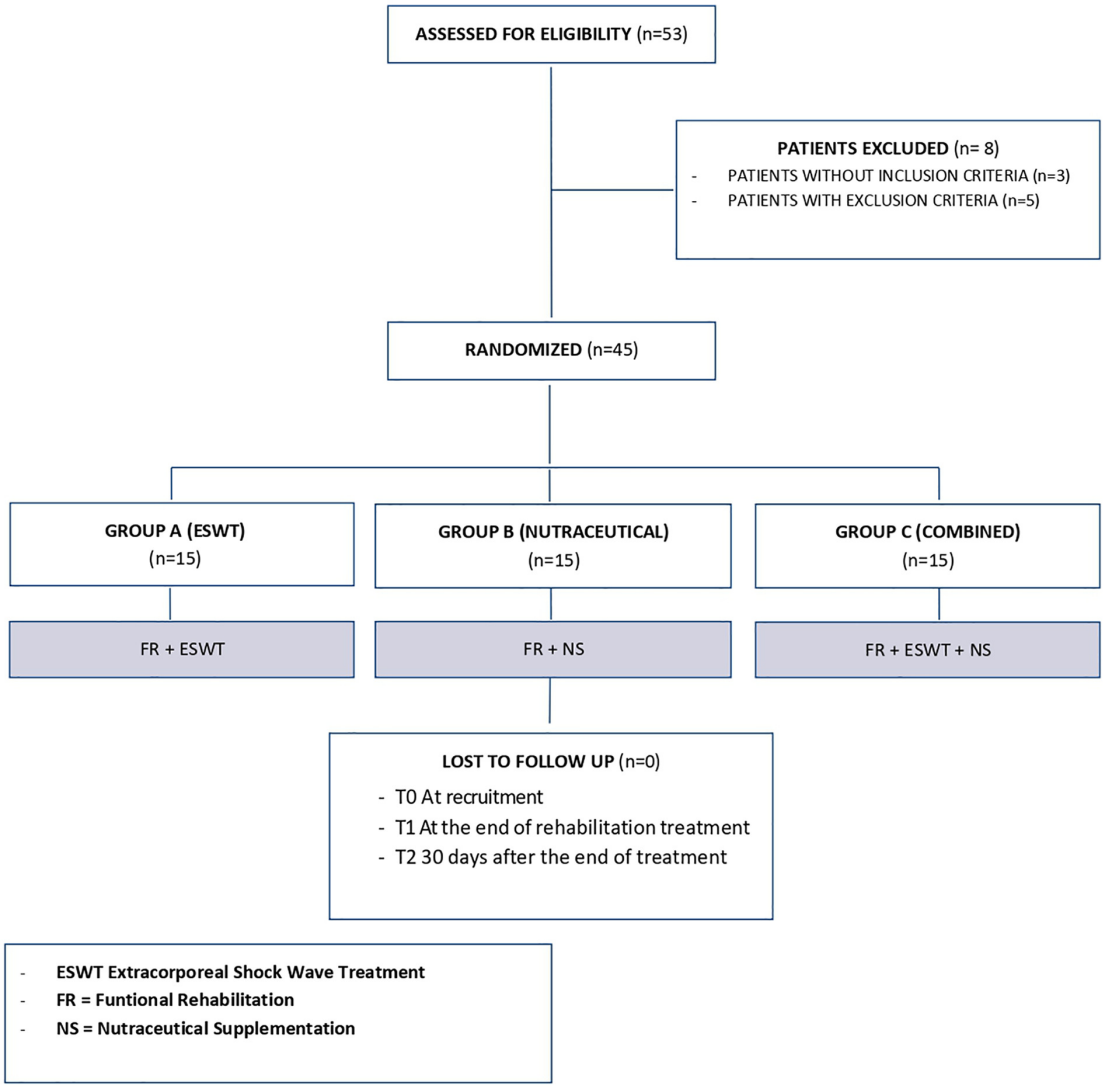
Study.

We enrolled 53 patients with LE; of these, 3 patients did not fit the inclusion criteria and another 5 had exclusion criteria instead, so 45 patients were included in the study (Figure 1). Participants were randomly divided into three groups of equal numbers. The baseline characteristics of the sample and initial assessment are summarized in Table 1, which shows the homogeneity of the three groups. The included patients had a mean age of 35.53 ± 7.55 years, with 24 men (53.3%) and 21 women (46.7%). At baseline, the mean NRS value was 6.02 ± 1.08, the mean PRTEE score was of 32.09 ± 5.23, and the mean Handgrip values were 32 ± 1.96 kg for men, and 18.9 ± 1.97 kg for women. The mean CET thickness was 5.24 ± 0.2 mm. The side of involvement (right 75.6% - left 24.4%) and upper limb dominance (right 82.2% - left 17.8%) were also recorded. No statistically significant differences were found among groups (Table 1).

**Table 1:** Demographic characteristics of the groups.

Variable	TOTAL (*n* = 45)	ESWT *n* = 15)	NS (*n* = 15)	ESWT+NS (*n* = 15)	*P*-Value
Age (year)	35.53 ± 7.55	35.46 ± 7.79	35.2 ± 7.32	35.46 ± 7.39	0.99
Sex					
Male	24 (53.3%)	8 (53.3%)	7 (46.7%)	9 (60%)	>0,05
Female	21 (46.7%)	7 (46.7%)	8 (53.3%)	6 (30%)	
Body mass index (BMI) (kg/m^2^)	25.81 ± 1.61	25.65 ± 1.4	26.02 ± 1.79	25.77 ± 1.69	0.75
Dominant hand					
Right	37 (82.2%)	12 (80%)	12 (80%)	13 (86.7%)	>0,05
Left	8 (17.8%)	3 (20%)	3 (20%)	2 (13.3%)	
Side of involvement					
Right	34 (75.6%)	11 (73.3%)	11 (73.3%)	12 (80%)	>0.05
Left	11 (24.4%)	4 (26.7%)	4 (26.7%)	3 (20%)	
NRS T0	6.02 ± 1.08	5.8 ± 1.15	6.1 ± 1.07	6.26 ± 1.03	0.50
PRTEE T0	32.09 ± 5.23	31.86 ± 5.99	32.27 ± 4.71	32.13 ± 5.27	0.97
HANDGRIP T0	M 32 ± 1.96 F 18.9 ± 1.97	M 32.62 ± 2.07 F 20.14 ± 2.03	M 31.57 ± 1.27 F 18.37 ± 2.07	M 31.56 ± 2.19 F 18.17 ± 1.17	0.45 0,12
CET THICKNESS T0	5.24 ± 0.2	5.25 ± 0.23	5.3 ± 0.19	5.27 ± 0.19	0.83

NRS, numerical evaluation scale; PRTEE, patient-rated tennis elbow evaluation; CET, common extensor tendon; FR, funtional rehabilitation; ESWT, extracorporeal shock wave treatment; NS, nutraceutical supplementation.

Table 2 shows the results obtained in group A at T1 and T2. At T1 no statistically significant differences were found for all the outcomes considered. At T2, however, there was a modest, but significant improvement in the NRS values (2.73 ± 0.8; ≤0.05) and the PRTEE Scale (16.8 ± 3.21; ≤0.05), as well as a reduction in CET thickness (4.95 ± 0.17; ≤0.05) (Table 2).

**Table 2:** Effect of treatment with FR + focal ESWT in the A group.

Characteristics	T0	T1	*p*-value	T2	*p*-value
NRS mean ± SD	5.8 ± 1.15	5.13 ± 1.06	0.11	2.73 ± 0.8	0.0001
PRTEE mean ± SD	31.86 ± 5.99	29.4 ± 5.77	0.26	16.8 ± 3.21	0.0001
Handgrip mean ± SD	M 32.62 ± 2.07 F 20.14 ± 2.03	M 33.37 ± 2.67 F 21.57 ± 2.51	0.54 0.26	M 34.5 ± 2.62 F 22.57 ± 2.82	0.41 0.49
CET Thickness mean ± SD	5.25 ± 0.23	5.17 ± 0.23	0.38	4.95 ± 0.17	0.005

NRS, numerical evaluation scale; PRTEE, patient-rated tennis elbow evaluation; CET, common extensor tendon; FR, funtional rehabilitation; ESWT, extracorporeal shock wave treatment.

Table 3 shows the effects of the combination of therapeutic exercise and nutraceutical supplementation in group B, at 15 days of treatment (T1) and at the end of therapy (T2). Also in this group, statistically significant improvements were recorded regarding the values at T2 of NRS (1.93 ± 0.6; ≤0.05), PRTEE scale (13.73 ± 4.38; ≤0.05) and CET thickness (4.55 ± 0.14; ≤0.05). However, in this group, there was a statistically significant improvement in pain symptoms already at T1 with an average NRS value of 3.8 ± 0.86; ≤0.05 (Table 3).

**Table 3:** Effect of treatment with FR + NS in the B group.

Characteristics	T0	T1	*p*-value	T2	*p*-value
NRS mean ± SD	6 ± 1.07	3.8 ± 0.86	0.00001	1.93 ± 0.6	0.00001
PRTEE mean ± SD	32.27 ± 4.71	28.6 ± 5.18	0.52	13.73 ± 4.38	0.00001
Handgrip mean ± SD	M 31.57 ± 1.27 F 18.37 ± 2.07	M 32.57 ± 1.271 F 19.75 ± 1.38	0.16 0.14	M 33.57 ± 1.4 F 21.87 ± 1.45	0.18 0.23
CET Thickness mean ± SD	5.3 ± 0.19	5.24 ± 0.18	0.42	4.55 ± 0.14	0.00001

NRS, numerical evaluation scale; PRTEE, patient-rated tennis elbow evaluation; CET, common extensor tendon; FR, funtional rehabilitation; NS, nutraceutical supplementation.

Table 4 shows the results obtained in group C at T1 and T2. At 15 days of treatment (T1) there were no statistically significant results for the outcomes investigated. At the end of the treatment (T2), however, statistically significant results were recorded in terms of reduction in pain with reduction in NRS values (1.4 ± 0.51; ≤0.05), improvement in function (PRTEE 10.33 ± 4.4; ≤0.05) and grip strength (handgrip males 51.44 ± 2.19 – females 31.33 ± 1.63; ≤0.05). There was also a significant reduction in CET thickness (4.51 ± 0.16; ≤0.05). (Table 4).

**Table 4:** Effect of treatment with focal FR + focal ESWT+NS in the C group.

Characteristics	T0	T1	*p*-value	T2	*p*-value
NRS mean ± SD	6.26 ± 1.03	5.33 ± 0.81	0.19	1.4 ± 0.51	0.00001
PRTEE mean ± SD	32.13 ± 5.27	29.33 ± 5.19	0.15	10.33 ± 4.4	0.00001
Handgrip mean ± SD	M 31.56 ± 2.19 F 18.17 ± 1.17	M 32.89 ± 1.96 F 18.67 ± 0.82	0.19 0.41	M 51.44 ± 2.19 F 31.33 ± 1.63	0.00001
CET Thickness mean ± SD	5.27 ± 0.19	5.19 ± 0.19	0.25	4.51 ± 0.16	0.00001

NRS, numerical evaluation scale; PRTEE, patient-rated tennis elbow evaluation scale; CET, common extensor tendon; FR, funtional rehabilitation; ESWT, extracorporeal shock wave treatment; NS, nutraceutical supplementation.

**Figure 2:**
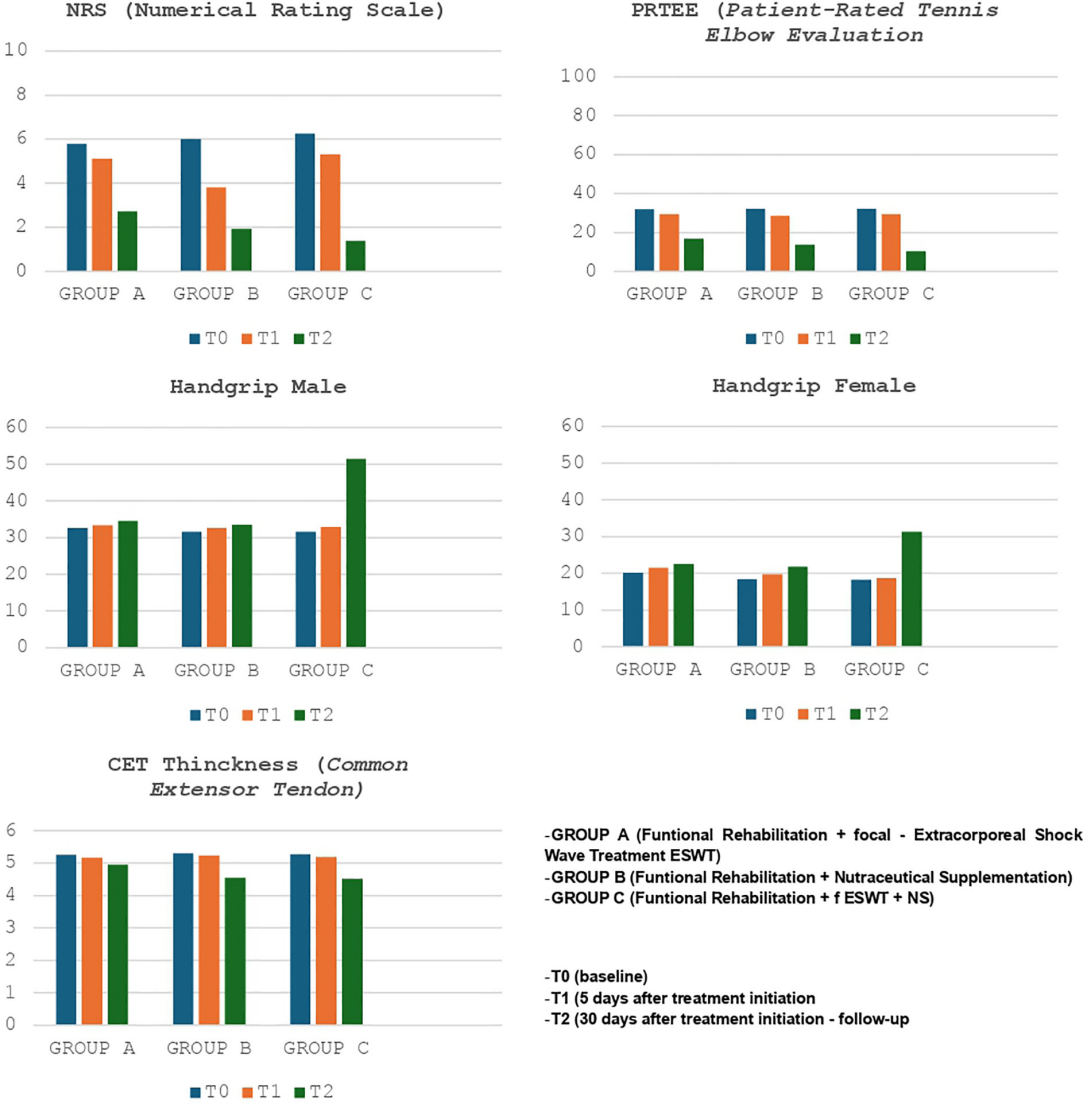
NRS, PRTEE, CET and hand grip female/male of Group A-B-C at T0-T1-T2.

Finally, we compared the results of the various groups at T0 and T2 (Figure 2 - Tables 5a-5b); substantial differences emerged in comparative analysis. Indeed, patients treated with therapeutic exercise, focal ESWT and nutraceutical supplementation (Group C) obtained statistically significant better results, compared to patients in groups A and B, in terms of reduction of both pain (F ratio- value 16.24 - *p* ≤ 0.05) and disability (F ratio-value 9.62 - *p* ≤ 0.05). Furthermore, at the end of the treatment (T2), the patients in group C had a significant improvement in their grip strength compared to the other two groups (Male F ratio-value 182.46 - *p* ≤ 0.05 - Female F ratio-value 51.14 - *p* ≤ 0.05) (Tables 5a-5b). None of the participants dropped out of the study before the scheduled end date or experienced adverse reactions to the proposed treatments.

**Table 5:** (a-b) *post hoc* tukey HSD comparison between the ESWT (group A), nutraceutical Supplementation (group B) and combined Treatment (group C) T0 and T2.

Characteristics	T0					T2				
	Group A	Group B	Group C	F-ratio Value	*p*- Value	Group A	Group B	Group C	F-ratio Value	*p*-Value
NRS mean ± SD	5.8 ± 1.15	6 ± 1.07	6.26 ± 1.03	0.7	0.50	2.73 ± 0.8	1.93 ± 0.6	1.4 ± 0.51	16.24	<0.05
PRTEE Scale mean ± SD	31.86 ± 5.99	32.27 ± 4.71	32.13 ± 5.27	0.02	0.97	16.8 ± 3.21	13.73 ± 4.38	10.33 ± 4.4	9.62	<0.05
Hanhgrip strength mean ± SD	M 32.62 ± 2.07 F 20.14 ± 2.03	M 31.57 ± 1.27 F 18.37 ± 2.07	M 31.56 ± 2.19 F 18.17 ± 1.17	0.81 2.37	0.45 0.12	M 34.5 ± 2.62 F 22.57 ± 2.82	M 33.57 ± 1.4 F 21.87 ± 1.45	M 51.44 ± 2.19 F 31.33 ± 1.63	182.46 51.14	<0.05 <0.05
CET (mm) mean ± SD	5.25 ± 0.23	5.3 ± 0.19	5.27 ± 0.19	0.18	0.83	4.95 ± 0.17	4.55 ± 0.14	4.51 ± 0.16	35.46	<0.05

NRS	Q	*p*-value
Group A Vs Group B	4.80	<0.05
Group A Vs Group C	8.01	<0.05
Group B Vs Group C	3.20	0.07
PRTEE	Q	*p*-value
Group A Vs Group B	2.94	0.10
Group A Vs Group C	6.20	<0.05
Group B Vs Group C	3.26	0.06
CET	Q	*p*-value
Group A Vs Group B	9.84	<0.05
Group A Vs Group C	10.73	<0.05
Group B Vs Group C	0.89	0.80
HANDGRIP M	Q	*p*-value
Group A Vs Group B	1.21	0.67
Group A Vs Group C	22.08	<0.05
Group B Vs Group C	23.29	<0.05
HANDGRIP F	Q	*p*-value
Group A Vs Group B	2.51	0.20
Group A Vs Group C	11.28	<0.05
Group B Vs Group C	13.79	<0.05

Post HOC Tukey HSD comparison between A- B C Groups.

NRS, numerical evaluation scale; PRTEE, patient-rated tennis elbow evaluation scale; CET, common extensor tendon.

## 4 Discussion

Based on the results of our study, we can state that the combination of therapeutic exercise, nutraceutical supplementation, and ESWT therapy is a viable treatment option for lateral epicondylitis. Indeed, patients benefit in terms of pain reduction and increased function, which correlates with improved ultrasonographic imaging. Therefore, in clinical practice, when we are faced with patients presenting with a picture of lateral epicondylitis, diagnosed clinically and by ultrasound examination, after careful evaluation by means of specific scales, we could set up a combined treatment to counteract the inflammatory process and restore function, so as to improve the patients' quality of life and make them independent in ADLs in the short term.

The treatment of LE is inherently multimodal. Pharmacological interventions, such as anti—inflammatory drugs, local anesthetics, and opioids are frequently used (32–34), often in association with rehabilitation treatments involving exercise, particularly stretching and eccentric strengthening (36). The combined effect of pharmacotherapy, therapeutic exercise, and physical modalities, such as ESWT, ultrasound (US), and high-intensity laser therapy (HILT) appears to be the most effective treatment strategy currently available (42, 44, 46). However, literature presents a non-uniform approach to LE treatment, as different studies explore various therapeutic options.

Several authors have evaluated the effects of conservative therapies. In agreement with our findings, de Sire et al. and Letizia Mauro et al. emphasized the importance of pain management in acute musculoskeletal conditions using appropriate pharmacological and rehabilitation therapies (66, 67).

Campos et al. and Uttamchandani et al. conducted reviews on conservative therapies, including therapeutic exercise, pharmacological, and physical therapies, demonstrating that most patients experience improved quality of life, pain reduction, and short-term gains in muscle function and strength (28, 68). Marigi et al. and Karabinov et al. also analyzed conservative treatment options, supporting their effectiveness in LE recovery but without examining the synergistic effects of combined therapies (69, 70).

The role of supplementary therapy in epicondylitis management has also been investigated. Tarpada SP et al. and Vitale et al. concluded that nutraceuticals containing collagen and hyaluronic acid, administered orally or via injection, effectively support physical and rehabilitation therapies and should play a primary role in epicondylitis treatment (3, 33). Pellegrino et al. studied the combination of hyaluronic acid injections with HILT laser therapy, demonstrating its superiority over therapeutic exercise alone in terms of muscle strength and function recovery (71). Several studies have assessed the effectiveness of individual physical therapies. Dolibog et al. examined the impact of electrostimulation on pain reduction (72), while Elsayed et al. compared the effects of HILT laser and ultrasound (73). Consistent with our findings, numerous studies have highlighted the benefits of ESWT in epicondylitis management (74). Pellegrino et al. compared focal and radial ESWT, outlining their differences in pain relief and functional improvement (75). Other studies have compared ESWT with alternative therapies. Cheema et al. examined the benefits of transcutaneous electrical nerve stimulation (TENS) vs. ESWT for pain relief, finding electroanalgesia superior due to ESWT's painful nature (76). Ozmen et al. compared ultrasound and ESWT, concluding that both are effective but neither is superior (77). Laser therapy has also been widely compared with ESWT. Sen et al. and Karaca et al. conducted randomized controlled studies demonstrating that laser therapy surpasses ESWT in pain reductiones (78, 79). ESWT has also been compared with corticosteroid (CS) injections (80) and PRP therapy, revealing comparable benefits (81, 82). Another treatment increasingly in vogue in recent times in the management of lateral epicondylitis are interventional ultrasound-guided procedures; this is a viable alternative that reduces painful symptoms and improves upper limb function when conventional physical therapies fail to resolve the issue. There are several products that can be injected at the tendon or peritendinous level: for example, corticosteroids, but especially compounds based on collagen and hyaluronic acid have been seen to lead to excellent results in terms of improved function and recovery of autonomy in ADLs. Of paramount importance is the use of ultrasound: first, for a better understanding of the pathology (and thus for better clinical decision making regarding even the eventual interventional procedure); second, because it provides a precise goal/guidance during the procedure, which is tailored to each patient (83). Less common therapies, such as peloidotherapy (studied by Koru et al. (84) and prolotherapy (examine by Ahadi T et al. (85), have also been explored, demonstrating effectiveness in LE treatment, albeit to a lesser extent.

Based on our findings and existing literature, no previous studies have compared the combination of ESWT and nutraceutical supplementation with therapeutic exercise. Another strength of this study is the adequate sample size across three treatment groups and the use of multiple rating scales alongside rigorous statistical analysis. However, future research should focus on larger patient populations to validate these findings. Our study has several limitations; it's an unblinded study so the results may not be easily generalizable to other populations. A control group with a single treatment (nutraceutical supplementation or exercise) was not included in the study to allow a clearer comparison of the effects of the intervention, as exercise was prescribed indiscriminately to all three groups. Another limiting aspect was the 30-day follow-up; certainly it would be desirable to perform clinical and ultrasound evaluation at 3 and 6 months, given the high frequency of recurrence and chronicity of lateral epicondylitis. Finally, it was not possible to find other numerical parameters, besides the CET thickness, that would take into account, for example, the reduction of vascularization and degeneration of the tendon analyzed. It would be also desirable for future research to continue to follow patients over time, and to evaluate any flare-ups by means not only of the objective examination, but also with ultrasound evaluations showing any reappearance of inflammatory phenomena and pictures of tendinosis. One could also correlate and/or compare our proposed treatment with the use of intra- and peritendinous corticosteroid or hyaluronic acid and collagen infiltrations.

## 5 Conclusions

Treatment of LE is multimodal and, in most cases, conservative. The use of focal ESWT and nutraceutical supplementation, associated with therapeutic exercise, might be a valid option for managing LE in terms of pain reduction, recovery of functional capabilities and reduction of the inflammatory state. Treatment with therapeutic exercise and nutraceutical supplementation (Group B), unlike the other two groups, proved beneficial in reducing pain as early as 15 days after starting therapy (T1). The combination of treatments (Group C), on the other hand, not only achieved superior results in pain reduction and functional recovery but was also the only approach that effectively restored grip strength at the end of treatment (T2). Therefore, the combined treatment of ESWT and nutraceutical supplementation (including hyaluronic acid, collagen, vitamin C, and manganese), along with therapeutic exercise, appears to have a synergizing effect, making it preferable to individual treatments. From the analysis of the results that emerged furthermore, we can state that statistical significance correlates with clinical significance, as the primary outcomes assessed showed significantly higher values than the calculated MCIDs. For NRS values in fact, values greater than 2.8 were obtained in all three groups at T2, as well as for the PRTTE Scale, in which there were improvements exceeding 11 points after 30 days of treatment. Regarding grip strength only Group C achieved the calculated MCID values in both males and females. Finally, a clinically significant reduction was seen in Groups B and C for common extensor tendon thickness values. The proposed therapies proved effective, safe and well tolerated among the patients in our study. Future studies should aim to compare the effectiveness of these treatments across larger patient samples.

## Data Availability

The original contributions presented in the study are included in the article/Supplementary Material, further inquiries can be directed to the corresponding author.
